# Adherence to antihypertensive medication in Russia: a scoping review of studies on levels, determinants and intervention strategies published between 2000 and 2017

**DOI:** 10.1186/s13690-019-0366-9

**Published:** 2019-09-25

**Authors:** Elena Viktorovna Bochkareva, Ekaterina Kronidovna Butina, Irina Vitalievna Kim, Anna Vasilievna Kontsevaya, Oxana Mikhailovna Drapkina, David Leon, Martin McKee

**Affiliations:** 1 Laboratory of Drug Prevention in Primary Healthcare, Federal State Institution “National Medical Research Center for Preventive Medicine”, Petroverigsky per., building 10/3, Moscow, 101990 Russia; 2 London School of Hygiene and Tropical Medicine, London, UK; 3 UiT, Arctic University of Norway, Tromso, Norway

**Keywords:** Hypertension, Medication adherence, Scoping review

## Abstract

**Background:**

Arterial hypertension (HT) is common in the Russian adult population, with half of affected individuals inadequately controlled. Low adherence to medication seems likely to be a factor. We report a scoping review of studies on adherence to antihypertensive therapy (AHT) in Russia to determine the extent of research undertaken, the frequency of adherence among adults diagnosed with HT, methodologies used in the studies, and their ability to describe determinants of adherence.

**Methods:**

A scoping review of published studies that have assessed adherence to AHT in Russian HT patients searched the main Russian and international electronic databases eLIBRARY.ru, Russian Medicine, Embase, MEDLINE for full-text reports published in the Russian language between 2000 and 2017. The last search was on November 28, 2017. Among 520 reports identified, 31 were included in the review.

**Results:**

Eighteen studies assessed adherence using the 4-item Morisky Medication Adherence Scale (MMAS-4); others used bespoke questionnaires or pill counts. 25 studies assessed levels of adherence, 11 examined its determinants, and 18 examined intervention strategies. The proportion of “adherent” patients varied from 11 to 44% using the MMAS-4, from 23 to 74% when using bespoke questionnaires, and from 5 to 43% when using pill counts. Adherence was associated with sociodemographic factors, access to free drugs provided through the Medicine Assistance Scheme (MAS), use of home blood pressure (BP) monitoring, anxiety, and comorbidity. There was no evidence that adherence was associated with income or physical activity. Evidence of an association between MAS, grade of HT, or experience of hypertensive crisis was inconclusive. Various methods to improve adherence were studied including patient education (improved from 1.8 to 3.9 points, *p* = 0.0002 or 2.80 to 3.79 points, *p* < 0.0001 measured by the MMAS-4), telephone reminders (p < 0.0001), training in home BP monitoring (*p* < 0.05), and use of fixed-dose combinations (p < 0.05).

**Conclusions:**

The main determinants of adherence to AHT are sociodemographic characteristics, the severity of HT, and presence of comorbidity. Patient education and use of fixed-dose combinations of drugs were identified as most important for improving adherence. Most studies assessing adherence use self-reported methods so there is a need for greater use of objective methods.

**Trial registration:**

This scoping review has not been registered.

**Electronic supplementary material:**

The online version of this article (10.1186/s13690-019-0366-9) contains supplementary material, which is available to authorized users.

## Background

Russia has one of the highest mortality rates from circulatory diseases in the world. In 2015 the age-standardized death rate was 368.8 per 100.000 [1], 2.5–4 times higher than in West European countries [1]. Arterial hypertension (HT) is among the main risk factors [2], affecting an estimated 44% of the Russian adult population [3], with only 53% of those with HT being controlled [3].

One reason for poor blood pressure (BP) control is thought to be inadequate adherence to treatment, [4] a substantial problem everywhere. A review of 21 clinical studies conducted outside Russia found that adherence to antihypertensive therapy (AHT) falls with time from diagnosis, with about half of patients discontinuing treatment after one year [5]. This poor adherence is, as expected, associated with treatment failure and adverse cardiovascular events [6] while good adherence has been linked to fewer adverse cardiovascular outcomes [7].

The scale of the problem means that much research has been undertaken to identify factors associated with poor adherence and to develop measures to improve it. However, while some of the conclusions from this work are generalizable across countries, it is important to take account of context, as there may be differences in health beliefs (such as understanding of the importance of continuing treatment indefinitely for an asymptomatic condition), health systems (such as how medicines are paid for, and other circumstances). Moreover, context-specific evidence is more likely to be accepted by national policy makers.

Here we report a scoping review of all Russian language studies presented in full-text reports on the problem of adherence to antihypertensive medication in the Russian population, the factors associated with adherence, interventions to improve adherence to treatment, and their effectiveness. This makes two distinct contributions. First, it provides the most detailed and comprehensive overview of what is known from the published literature about this important issue in Russia, summarizing the often neglected corpus of work published in the Russian language. Second, we have summarized the results of research in the quantitative indicators and described of the revealed patterns.

As many readers will be unfamiliar with the Russian health system, we summarize the key elements of medicines supplies in Table 1.

**Table 1:** Pharmaceuticals in the Russian health system

State medical institutions in the Russian Federation provide free medical treatment to all in-patients but, after discharge, patients must pay the full cost unless they are in one of the groups entitled to free medications or at a 50% discount, as set out in a law from 1994. These include children in large families who are under a certain age (3 or 6 depending on family size), those receiving the minimum pension, invalids, veterans of the Great Patriotic War and other military operations, and those involved in the Chernobyl disaster. Entitlement extends to immediate family members. Since 2008, those in these categories can choose an alternative, whereby they receive monetary benefits instead. In practice, a growing number of the 19 million potential beneficiaries choose monetary benefits, leaving less than 4 million receiving. This can be explained by how free and subsidized medicines are available only in certain pharmacies in specific medical institutions and a widespread belief that essential drugs are often unavailable in these pharmacies. Those choosing monetary compensation can thus obtain their medicines from private pharmacies, albeit at additional cost. A recent study of medicines availability and affordability in state and private pharmacies in six Russian citizens did, however, find that common cardiovascular medicines were widely available and, in private pharmacies, reasonably affordable. However, where state pharmacies stocked generic versions, they did not also stock branded equivalents [8].	
Information on prescribing for hypertension in Russia can be found in the RELIF III study. The most frequent classes of drugs were angiotensin-converting-enzyme inhibitors (78%), diuretics (40%), beta-blockers (36%), and calcium antagonists (19%). The authors reported that angiotensin-converting-enzyme inhibitors were more likely to be taken regularly, specifically Prestarium, Renitec, and Hartil [9].	

### Research questions

We address the following questions by means of a scoping review of the Russian language literature pertaining to studies of adherence to antihypertensive medications conducted in Russia:

1. What levels of adherence are found among adults diagnosed as hypertensive?
2. What sociodemographic and clinical factors are associated with adherence?
3. What robust evidence has been generated as to effective interventions used in Russia to increase adherence to treatment?

## Methods

This scoping review was reported in accordance with the reporting guidance provided in the Preferred Reporting Items for Systematic reviews and Meta-Analyses statement extension for Scoping Reviews (PRISMA-ScR) [10] (Additional file 1).

ScR protocol was not published.

### Eligibility criteria

Reports were included in this scoping review if they met the following criteria:

- Target population included the Russian adult population aged 18 years and over with a diagnosis of HT defined as BP ≥140/90 mmHg and/or taking regular antihypertensive medication;
- Articles that report on adherence to AHT, regardless of how adherence is measured;
- Types of study designs - randomized controlled trial studies, non-randomized trial studies, observational studies (cross-sectional, cohort studies), registers;
- Reports published in the Russian language;
- Full-text original reports;
- Published in journals or proceedings of conferences;
- Literature published from January 2000 to November 2017. The last search was conducted in November 28, 2017.

There was no restriction on duration of treatment or sample size. There were no restrictions on interventions, comparators, and outcomes.

### Information sources

Searches of the main Russian and international electronic databases were complemented by iterative searches using Internet search engines, personal contact with Russian experts working on HT, and queries to authors of identified studies by phone or e-mail (also used where clarification was sought about survey methods, instruments (such as the name of the questionnaire) and duration of observation). Reference lists were also searched. This comprehensive approach was taken to reduce potential bias by including only easy-to-locate studies that may have larger effect sizes due to publication bias.

The Russian databases were:

- eLIBRARY.ru - the largest Russian information portal (https://elibrary.ru/)
- Central Scientific Medical Library “Russian Medicine” - second in size Russian medical information portal (http://www.scsml.rssi.ru/)

The international databases were:

- Embase (https://www.elsevier.com/solutions/embase-biomedical-research)
- MEDLINE (PubMed) (https://www.ncbi.nlm.nih.gov/pubmed)

The search for sources was conducted between October 2017 and November 2017 (last date searched).

For eLIBRARY.ru 333 publications were obtained by October 17, 2017.

For Russian Medicine 69 publications were obtained by October 20, 2017.

For Embase 87 publications were obtained by November 10, 2017.

For MEDLINE (PubMed) 31 publications were obtained by November 28, 2017.

The personal contacts with authors to identify additional sources were conducted from December 2017 to January 2018.

If more than one publication related to the same study, all were used to provide as much information as possible.

Full search strategies specific to the different databases are provided in Additional file 2.

### Study selection

Having eliminated duplicates, titles and abstracts were reviewed by 2 researchers to assess eligibility, with differences resolved by discussion or, where necessary, by consultation with a third team member. Those not available electronically were obtained as hard copies. Those potentially eligible were read by each researcher to confirm eligibility and those retained were categorized to covering one or both of the following two areas:

- Levels, patterns and determinants of adherence
- Interventions to improve adherence, including evaluations of effectiveness

Each paper was read three times by a different team member who extracted the key findings.

### Data charting process

Authors created a matrix (an Excel spreadsheet) to chart relevant information about all the sources reviewed. Specifically, the chart included details about the authors, year of publication, study setting, population/participant selection criteria, study design, sample size, age of participants, HT grade, nature of intervention, adherence measure, factor associated with adherence and main results (Table 2, Additional files 3, 4 and 5). Matrix was piloted with five papers and adapted in the light of this experience.

**Table 2:** Characteristics of included studies on adherence to antihypertensive therapy in adult population in Russia from 2000 to 2017

Reference	Year of publication	Study setting	Participant selection criteria	Design	Sample size	Age (years)	HT grade^a^	Critical appraisal/quality assessment of findings (a) Strengths (b) Weaknesses
Ageev et al. [11]	2008	Patients who visited outpatient department of the Russian Cardiology Scientific and Production Center	Men or women older than 18, with SBP 140–179 mmHg, DBP 99–100 mmHg, high cardiovascular risk, not taking of ACE inhibitors and diuretics, without secondary HT, heart failure, renal and hepatic impairment, insulin-treated DM. Recruitment process not described	Randomized non-blinded controlled intervention study	60	62.5 ± 2.2	1–3	(a) prospective study, follow up period 6 mth; (b) small sample size, incorrect DBP level in inclusion criteria
Kobalava et al. [12, 13]	2011	Patients attending 240 cardiologists in 17 Regions	Men or women with uncontrolled HT, non-adherent, absence of contraindications to ACE inhibitors taking, no eligibility to receive MAS	Randomized non-blinded controlled intervention study	906	56.2 ± 10.6 (female)/ 54.9 ± 10.9 (male)	Uncontrolled HT^b^	(a) multicenter study, follow up period 12 mth, big sample size; (b) including only non-adherent patients
Sarycheva et al. [14]	2017	Single outpatient clinic in Moscow Region. 300 patients have been examined before 150 patients included	Men or women aged 40–65, with ineffective treatment of HT and dyslipidemia, SBP > 140 mmHg, DBP > 90 mmHg, without IHD, DM and other severe diseases	Randomized non-blinded controlled intervention study	150	40-65y	HT patients with high cardiovascular risk	(a) follow up period 12 mth; (b) there are no basic data of adherence
Fofanova et al. [15]	2008	Patients who visited outpatient department of the Russian Cardiology Scientific and Production Center	Men or women older than 18, with SBP 140–179 mmHg, DBP 99–100 mmHg, not taking of ACE inhibitors and diuretics, without secondary HT, heart failure, renal and hepatic impairment, insulin-treated DM. Recruitment process not described	Randomized non-blinded controlled intervention study	60	61.2 ± 1.8 (female)/ 61.8 ± 2.1 (male)	1–2	(a) patients with high and very high cardiovascular risk are included, for which adherence to therapy is particularly important, follow up period 6 mth; (b) small sample size, incorrect DBP level in inclusion criteria
Karpov et al. [16]	2013	Patients attending any of 700 cardiologists in 51 Regions, each recruiting 3 patients	Men or women older than 18, with uncontrolled HT on treatment. Recruitment process not described	Prospective observational intervention study	2120	22–88 y	2–3	(a) big sample size, multicenter study; (b) relatively short follow up period 3 mth and no control group
Glezer et al. [17]	2016	Patients attending 197 physicians in 48 Regions	Men or women aged 18–79, with essential HT, SBP ≥140 mmHg, DBP ≥90, but <110 mmHg	Prospective observational intervention study	940	56.5 ± 11.5	1–2	(a) big sample size, multicenter study; (b) relatively short follow up period 3 mth, no control group
Glezer et al. [18]	2015	Patients attending 243 physicians in 51 Regions	Men or women older than 18, with HT taking 2 or more antihypertensive drugs who have not reached their BP target, SBP 140–179 mmHg, DBP 90–109 mmHg, without contraindications to ACE inhibitors and calcium channel blockers	Prospective observational intervention study	1351 included, 1061 completed the protocol	59.4 ± 11.1	Essential HT	(a) big sample size, multicenter study; (b) relatively short follow up period 3 mth, no control group
Glezer et al. [19]	2016	Patients attending 442 physicians in 29 cities	Men or women older than 18, with HT on treatment who have not reached their BP target	Prospective observational intervention study	1969	60.1 ± 0.3	No data	(a) big sample size, multicenter study; (b) relatively short follow up period 3 mth, no control group
Kagramanyan [20]	2015	Not stated The author is affiliation at Yaroslavl State Medical University	Men or women aged 18–80, with grades 1–3 of HT, who visited the Municipal Clinical Hospital	Prospective observational intervention study	50	64.06 ± 0.49 (female)/ 61.88 ± 1.28 (male)	1–3	(a) studying of adherence in patients with 3 different socially significant nosologies - HT, asthma and alcohol abuse; (b) small sample size, large age range, the real number of HT patients is represented incorrectly
Kaskaeva et al. [21]	2015	Not stated	Male patients aged 20–64 with grades 1–3 of HT. Recruitment process not described	Non-randomized comparison of 3 groups	250	20–64 y (male)	1–3	(a) patients of employable age + relationship adherence to job; (b) described as randomized but groups selected on basis of employment: train drivers (112), other railway workers (50), non-railway workers (88)
Ushakova et al. [22]	2005	Regional cardiology clinic in Ivanovo city	Men or women with grade 2 of HT on treatment, without IHD and DM	Prospective observational intervention study	52	50.08 ± 7.25	2	(b) small sample size, no control group, patients with grade 2 of HT only included
Chazova et al. [23]	2014	Patients who visited outpatient department of the Russian Cardiology Scientific and Production Center	Recruitment process not described	Prospective observational intervention study	193	60.3 ± 8.0	No data	(a) scope of sessions with patients, duration of sessions and number of the studying patients in group corresponded to the standards approved by the Ministry of Health, it is important for working at outpatient care settings; (b) the control group is formed from abandoning the patient education, the number of patients in the control group is 2 times less than in the intervention group (65:128), short follow up period 6 weeks
Fofanova et al. [24]	2009	Patients attending 185 cardiologists in 84 policlinics of Moscow	Men or women with SBP 140–179 or DBP 99–100 mmHg, not taking calcium channel blockers	Cross-sectional	4816	62.2 ± 0.2	1–2	(a) big sample size; (b) incorrect DBP level in inclusion criteria, only possible to extract baseline data
Donirova et al. [25]	2012	Ambulatory care facility	Men or women with HT on treatment	Cross-sectional	74	18 y and older	No data	(b) small sample size (14 vs 60)
Loukianov et al. [26]	2017	Patients attending 185 physicians or cardiologists of the same from 3 randomly selected outpatient clinics of Ryazan and the Ryazan region in March–May 2012 (consecutive inclusion of all who applied from March 01 to May 27)	Patients older than 18, with combination of IHD, HT, chronic heart failure, permanent residence in the Ryazan and the Ryazan region	Register	2303	70.3 ± 10.7 (ppl with history of MI), 69.9 ± 11.0 (ppl without history of MI)	1–3	(a) collection of adherence data using MMAS-4 in a large outpatient register (b) all patients, irrespective of history of MI, had complex pathology of IHD, HT and chronic heart failure. Therefore it is impossible to estimate independent association between HT and adherence.
Fofanova et al. [27]	2014	Patients who visited outpatient department of the Russian Cardiology Scientific and Production Center	Men and women with HT and examined by psychiatrists	Cross-sectional	161	19–75 (female)/ 53.4 ± 11.4 (male)	1	(a) assessment of adherence and psychosomatic aspects; (b) groups selected on basis of adherence to treatment: low adh – 131 ppl, high adh – 30 ppl
Soboleva et al. [28]	2012	Regional clinical hospital and ambulatory care facility	Patients with grades 1–3 of HT and cardiovascular disease. Recruitment process not described.	Cross-sectional	242	18 y and older	1–3	(b) only possible to extract baseline data
Oganov et al. [29]	2007	Patients attending 512 physicians in 20 cities	Men or women with HT and/or IHD	Cross-sectional	2496	18 y and older	1–3	(a) big sample size; (b) no prospective stage
Olejnikov et al. [30]	2014	Not stated The authors are affiliation at Penza State Medical University	Men or women older than 60, with grades 1–2 of HT. Recruitment process not described	Cross-sectional	75	66.6 ± 4.7	1–2	(a) studying adherence in the elderly; (b) non-standard way of MMAS-4 analyze, small sample size, only possible to extract baseline data
Smirnova et al. [31]	2012	Ambulatory care facility	Patients aged 45–75, with grades 1–2 of HT. Recruitment process not described	Randomized non-blinded controlled intervention study	60	Intervention group: 62 ± 9.4, control group: 63 ± 8.9	1–2	(a) complex intervention on adherence; (b) small sample size, relatively short follow up period – 3 mth
Vologdina et al. [32]	2009	Not stated	Men and women with IHD and grades 1–2 of HT. Recruitment process not described	Randomized non-blinded controlled by closed envelope method	70	80.7 ± 2.7 (female)/ 80.3 ± 2.5 (male)	1–2	(a) studying adherence in the elderly; (b) small sample size, relatively short follow up period – 3 mth
Sviryaev et al. [33]	2006	Ambulatory care facility	Men or women older than 18, with grades 1–2 of HT with irregular therapy	Prospective observational intervention study	115	51.3 ± 9.6	1–2	(a) follow up period 6 mth; (b) no control group, numerical indicators of adherence level aren’t presented in the publication
Morozov et al. [34]	2010	The authors are affiliation at Russian military medical Academy, St. Petersburg	Patients with grades 1–2 of HT	Cross-sectional	86	30–73 y (54 ± 4,8)	1–2	(b) only possible to extract baseline data, non-standard way of MMAS-4 analyze
Kotovskaya et al. [35]	2015	Patients attending 830 physicians in 113 cities	Men or women older than 18, with uncontrolled HT taking ACE inhibitors or angiotensin receptor blockers	Prospective observational intervention study	2435	59.3 ± 11.2	Uncontrolled HT^b^	(a) big sample size, multicenter; (b) MMAS modified with 2 additional questions, no control group, relatively short follow up period – 3 mth
Panov et al. [36]	2015	Federal Medical Research Center, St. Petersburg	Patients with grades 1–2 of HT and IHD	Prospective observational intervention study	60	57.65 ± 1.59	1–2	(a) follow up period - 12 mth; (b) small sample size
Oschepkova et al. [37]	2004	Patients who visited outpatient department of the Russian Cardiology Scientific and Production Center	Men and women aged 30–71, with grades 1–2 of HT, without MI, stroke, heart failure, heart arrhythmias. Recruitment process not described	Randomized non-blinded controlled intervention study	30	54 ± 11	1–2	(a) home BP devices as a way to increase adherence; (b) described as randomized but main group – 19 ppl, control group − 11, small sample size
Kontsevaya et al. [38, 39]	2015	Patients who visited Outpatient Cardiology Clinic	Men or women with grades 1–3 of HT	Cross-sectional	1419	61.94 ± 0.26	1–3	(a) big sample size, a large number of factors associated with adherence: sociodemographic, clinical, etc.; (b) no prospective stage
Kopnina et al. [40]	2008	Not stated	Patients with HT. Recruitment process not described	Cross-sectional	30	51 ± 1.14 (female)	2	(b) small sample size, only women are included in the study
Sergeeva et al. [41]	2012	Patients of the cardiological and endocrinological department of the Regional Clinical Hospital	Men and women with HT or HT + DM. Recruitment process not described	Cross-sectional	190	With HT: 47.6 ± 0.4, with HT + DM: 44.7 ± 0.2	1–3	(a) association of adherence with hypertensive crisis was shown; (b) no data on validation of bespoke questionnaire

*ACE* inhibitors, angiotensin converting enzyme inhibitors, *CVD* cardiovascular diseases, *DBP* diastolic blood pressure, *DM* diabetes mellitus, *HT* arterial hypertension, *IHD* ischemic heart disease, *MAS* Medicine Assistance Scheme, *MI* myocardial infarction, *MMAS-4* 4-item Morisky Medication Adherence Scale, *mth* months, *ppl* people, *SBP* systolic blood pressure

^a^ Definitions of office blood pressure levels (mmHg): grade 1 hypertension: 140–159 and/or 90–99; grade 2 hypertension: 160–179 and/or 100–109; grade 3 hypertension: ≥180 and/or ≥ 110

^b^ Uncontrolled HT was defined with patients not taking a previously prescribed therapy, registered in the medical records or insufficiently effective therapy

### Data items

A range of variables were extracted from reports of the studies.

Article details such as title, first author, date of publication, year of distribution, publication status, region(s) in which the study was conducted, and institutional setting.

Study design: randomized or non-randomized trial, cohort study, case-control study, register-based. If the design was a trial, we collected additional information on allocation concealment. Study objective, study duration, and sample sizes were also extracted.

Definitions of adherence: type of adherence measure: 4-item Morisky Medication Adherence Scale (MMAS-4), pill counts, bespoke questionnaire, etc.; indicators of adherence: MMAS-4 score points, percentage achieving a score of 4, pill counts compliance, and percentage of adherent people etc. (Additional file 3).

Determinants of adherence were extracted. These included baseline sociodemographic characteristics of HT patients: sex, age, education level, marital status, employment status, income, living in a city; disability; clinical characteristics of patients: HT grade, duration of HT, physical activity; associated clinical conditions (for example, ischemic heart disease, history of myocardial infarction, hypertensive crisis, etc.); concomitant diseases (for example, diabetes mellitus, panic attacks, subclinical depression, etc.); other data on instruments used in surveys; pharmacological therapy: therapeutic category, INN and commercial name, drug administration schedule, dose, dosage form; eligibility for the Medicine Assistance Scheme (MAS) which provides free drugs for certain categories of patients; home BP device availability; and frequency visits to the doctor (Additional file 4).

#### Intervention characteristics

Examples of content extracted included specific strategies to address barriers to adherence: special packaging of medications (e.g. blister packs, pill boxes), amount of prescribed medications, eligibility for the MAS, interventions designed to improve communication with patients, including more frequent visits, motivational interviewing, patient education, home BP monitoring, and provision of written instructions etc. (Additional file 5); mode of delivery: face-to-face, telephone, internet etc.; professions involved: pharmacist, physician, etc.; duration and number of sessions/consultations; and any other types of interventions in the experimental group;

Other data recorded included the main results, BP dynamics, achievement of target BP levels, author’s conclusions, and any reason for excluding the article.

### Study quality assessment

In assessing the quality of study, we considered use of validated questionnaires, objective methods for adherence assessing, study design, presence of randomization, blinding, sample size, and follow-up as appropriate.

### Synthesis of results

We grouped the studies by study questions analyzed (adherence, determinants and interventions), and summarized the type of settings, populations and study designs for each question, along with the measures used and a summary of findings. The results of this scoping review were synthesized using both a numerical summary, outlining relevant data from the included studies, and a narrative synthesis interpreting the results.

## Results

**Fig. 1:**
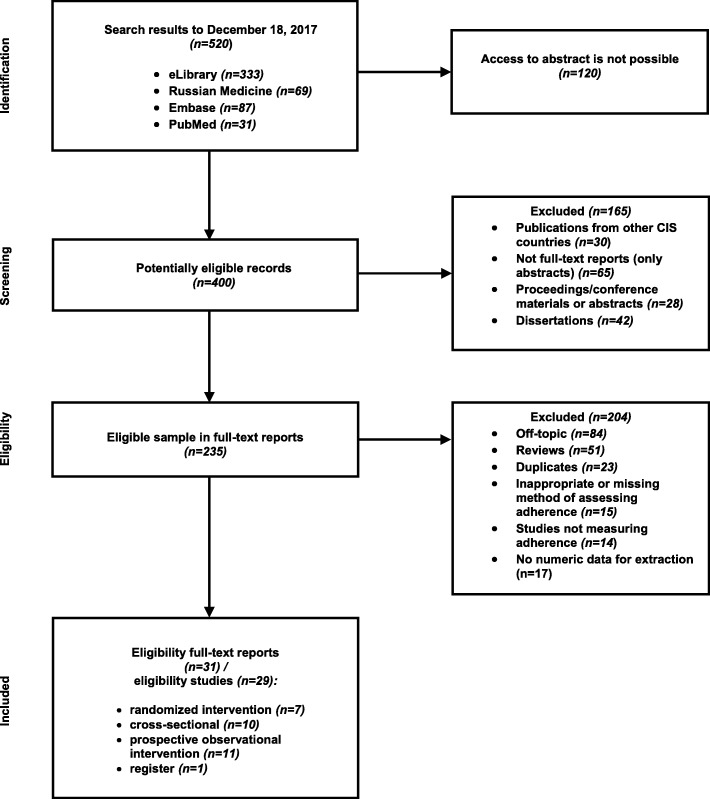
Flow diagram indicating the study selection process on adherence to antihypertensive therapy in adult population in Russia from 2000 to 2017

The initial search identified 520 references (Fig. 1). Of these 120 were excluded because it was not possible to obtain the abstract despite exhaustive searches. Most of these were published in regional journals unavailable in electronic format, with only tables of contents available online, printed in individual regions in small numbers.

The remaining 400 were screened against the inclusion criteria. The 235 potentially relevant reports were reviewed as full texts (eight were not available electronically, but three could be obtained as hard copies, leaving 5, from local journals that could not be obtained), leaving 31 eligible reports included in the review. The results from 2 studies were presented in 2 separate publications, resulting in us considering data from 29 individual studies.

The characteristics of the studies are presented in Table 2. Overall we included seven randomized intervention studies, ten cross-sectional studies, eleven prospective observational intervention studies (including six multicenter studies), and one report based on data from a register. None of the randomized studies were blinded. In four of the prospective observational intervention studies, it was only possible to extract baseline data as follow up data were not reported [24, 28, 30, 34]. Results from these studies were transformed into cross-sectional data.

The total number of patients in the studies selected for analysis was 23,127, with individual studies size ranging from 30 to 4816 participants.

The duration of follow-up varied from 6 weeks to 48 months.

### Adherence measurement

Adherence to AHT was assessed using MMAS-4 in 18 of the 29 studies. Ten studies used a bespoke questionnaire or a single question about regularity of taking drugs that was included in a questionnaire on a wider range of issues, supplemented with 3 clarifying questions. The bespoke instruments included pill counts in 6 studies; the MMAS-4 but analyzed in a non-standard way in 1 study [34], and the Morisky-Green questionnaire, modified with 2 additional questions in one study [35]. Several studies used more than one method, for example MMAS-4 and pill counts.

No studies used objective methods of assessing adherence - measurement of drugs in biological fluids in blood, urine, or indirect methods - electronic dispensers. The bespoke questionnaires were only available in Russian and there was no information about whether they had been validated.

Adherence was assessed using data from cross-sectional surveys and from baseline data in prospective observational intervention studies. In studies using the MMAS-4 [11–19, 21, 23–27, 30, 33] adherence to AHT varied from 1.62 ± 0.27 [11] to 3.12 ± 0.86 points [35] out of a maximum of four (Additional file 3). Among these studies, the highest rates of adherence were found in post-marketing studies, all with large sample sizes. Scores were 2.8 [19], 2.78 [16], 2.95 [18] and 3.12 ± 0.86 points [35]. In the studies reporting baseline adherence, the highest rates were in those that included a high proportion of patients with concomitant diseases: ischemic heart disease, chronic kidney disease, stroke, transient ischemic attack, diabetes mellitus, etc. [29], who can be expected to have a strong motivation to adhere to medication. The lowest baseline adherence rates, with scores of 1.62 [11] and 1.7 [31] were observed in studies with a small number of patients who only had HT.

Eleven studies measured the proportion of “adherent” patients, i.e. reporting 4 points on the MMAS-4. Results varied, from 11.1% [15] to 44.2% [35]. The highest proportion of “adherent” patients was again noted in a study where many subjects had concomitant diseases [35].

Adherence, as measured by bespoke questionnaires was reported in eight out of nine papers [20, 22, 28, 29, 37–41], one of the studies being published in two papers [38, 39], three studies reporting baseline adherence [20, 22, 37]. In two studies, 38.5 and 74.9% of patients took AHT daily [22, 38], in another 56% fully complied with medical recommendations, including the use of antihypertensive drugs [28], “high adherent”, “sufficiently adherent” or “regularly taken” according to the criteria set by the researchers varied from 23.3 to 60.5% [20, 29, 37, 40, 41]. However, these findings are difficult to compare with those from other studies because of lack of information on the instruments used.

Adherence using pill counts was examined in five studies [31–34, 36] but baseline adherence rates were reported in only 3, ranging from 5 [31] to 43% [32]. This large difference may reflect the small sample sized (60 and 115).

### Factors associated with adherence to antihypertensive therapy

12 studies examined associations between adherence and various socio-demographic, clinical and other variables (access to the MAS, home BP monitoring, frequency of visits to a doctor, etc.). Higher adherence was associated with female gender [29, 33, 35, 38, 41], age over 50 years [29, 33], not living alone [29, 38], employment [27, 33], higher education [33], and living in a city [41] (Additional file 4).

Higher adherence was associated with comorbidity, including: ischemic heart disease [24, 29, 38], history of myocardial infarction [26, 29], arrhythmias [27], diabetes mellitus [24, 29, 35], and psychiatric disorders [27]. High adherence was also associated with onset of HT at a young age [27] and use of fixed dose combination therapy [38].

The findings of studies of the association between adherence and grade of HT, experience of hypertensive crisis, and duration of HT were not consistent [27, 29, 33, 38–41].

There was no evidence that adherence was associated with income and physical activity [29].

#### Features of patient management in outpatient settings and adherence to therapy

Home BP monitoring [24, 29, 31] and more frequent visits to the doctor [34] were associated with better adherence. The association with eligibility for the MAS was also conflicting. In two studies [24, 39] it was associated with lower adherence [24] or failure to follow the recommended regimen [39] but in another, there was no association [38].

Taking multiple antihypertensive drugs (2 or more) was associated with decreased adherence [35, 39].

### Assessment the effectiveness of interventions aimed at increasing adherence

Interventions to increase adherence were mainly either patient education (in various forms) in 6 studies [12, 13, 19–23] or optimization of the drug administration regimen in 11 studies [11–13, 15–19, 32, 33, 35, 36], among which 9 used a fixed-dose combination of drugs [11, 15, 16, 18, 19, 32, 33, 35, 36]. One study gave patients an electronic version of the SCORE scale, with the physician showing the patient how their risk would be reduced if they stopped smoking, controlled their BP and reduced their cholesterol [14]. In two studies [31, 37] patients were trained in home BP monitoring, intended to increase adherence.

Several studies used more than one method to increase adherence.

#### Randomized controlled intervention studies

In one study [12], a multi-faceted intervention, which included information within an educational program for patients, a free first package of antihypertensive drugs, regular visits to the doctor, and telephone reminders, was associated with a significant improvement in adherence. After 12 months, the proportion of adherent patients who achieved an MMAS-4 of 4 points in the intervention group was 71.7%, up from 52.2%, in the control group. The difference at follow up was highly significant (*p* < 0.0001) (Additional file 5). Demonstration of an electronic version of the SCORE scale to patients, highlighting benefits of reducing cardiovascular risk by smoking cessation, BP control, and reduced cholesterol, was associated with higher adherence than in a control group, with scores at 6 months of 2.75 and 1.88 points (*p* < 0.001), respectively, and 2.14 and 1.27 points (p < 0.001), respectively after 12 months [14].

Training patients in home BP monitoring was associated with significant improvement in adherence, with the effect persisting at 1 year [37].

Provision of an automatic BP monitor was associated with a significant increase in adherence to AHT, with a MMAS-4 score increasing from 1.7 ± 1.2 to 3.0 ± 1.1 points, p < 0.000 in the intervention group and from 5 to 96.4% (p < 0.001) in the index of compliance [31].

The use of fixed-dose combinations was associated with better adherence to therapy in several studies [11, 15, 16, 18, 19, 32, 33, 35, 36], including 3 randomized non-blinded controlled trials [11, 15, 32]. Two of these used the MMAS-4 [11, 15] and the other a compliance index [32].

#### Non-randomized intervention studies

Five studies evaluated so-called “patient education” or similar interventions [19–23]. Those using the MMAS-4 reported increases from 2.80 to 3.79 points (*p* < 0.0001) [19] or from 1.8 to 3.9 points (*p* = 0.0002) [23], while the proportion of patients with an MMAS-4 of 4 points increased from 38.6 to 57.9% (*p* = 0.04) [21]. Adherence, as measured by a bespoke questionnaire improved from 27 to 67% (*p* < 0.05) [20]. The proportion of patients who reported measuring their BP daily increased from 28.8 to 65.4% (p < 0.05) and taking antihypertensive drugs daily increased from 38.5 to 82.7% (p < 0.05) [22].

The use of fixed-dose combinations of antihypertensive drugs was evaluated in 7 non-randomized studies [16–19, 33, 35, 36]. As in the randomized trial studies, fixed-dose combinations were associated with significant increases in adherence, as measured by the MMAS-4 compared to baseline [16–19, 33, 35].

Using bespoke questionnaires, consistently high adherence was observed during the first year of treatment with a fixed-dose combination of an angiotensin converting enzyme inhibitor and calcium channel blocker, at 97 and 93% at 6 and 12 months respectively [36].

### Critical appraisal/study quality assessment

Problems included the small number of randomized studies none of which were blinded, heterogeneity of patient groups in non-randomized studies, small sample size in some studies, heterogeneity of samples, presence of concomitant pathology, use of subjective methods of assessing adherence (questionnaires), and incomplete presentation or selective reporting of results [42].

## Discussion

This is the first scoping review of Russian language studies on adherence to AHT. Our search strategy was designed to include as many primary publications as possible, although it was concerning that abstracts for a large number of studies could not be located. This highlights an issue that has not, to our knowledge, received adequate attention so far. There are a large number of regional medical journals, printed in small numbers, and while their tables of contents are available electronically, their content (including abstracts) is not. There is no central repository. While, in theory, it might be possible to obtain copies from publishers, the logistical barriers would be formidable and, given the methodological weaknesses reporting in many of the papers obtained, unlikely to be commensurate with the information that might be extracted from them.

In accordance with the questions and objectives in this scoping review, the key findings are as follows. Adherence was assessed using MMAS-4 in 18 studies and in other studies using bespoke questionnaires. In the Russian population, the baseline MMAS-4 scores varied from 1.62 ± 0.27 points [11] to 3.12 ± 0.86 points [35]. The proportion of patients with 4 points on the MMAS-4 varied from 11.1% [15] to 44.2% [35], while in studies using a bespoke questionnaire, the frequency of adherence varied from 23.3% [40] to 74.9% [38]. The latter were patients attending an outpatient cardiology clinic. Relatively low levels of adherence were observed in most studies [11, 20, 22, 23, 31, 32, 37, 40, 41]. These findings are consistent with a cross-sectional study by Cybulsky et al. on 1068 working-age men in Izhevsk, Russia, which reported 41% of patients taking antihypertensive drugs daily [43].

Many studies included quite large samples, from several hundred to several thousand people [12, 13, 16–19, 24, 26, 29, 35, 38, 39], but some were much smaller, from 30 to 75 people [11, 15, 20, 22, 25, 30–32, 36, 37, 40]. The follow up period varied from 6 weeks [23] to 12 months [12, 13, 36, 37]. One study lasted 4 years [16], but follow up data were unavailable for analysis.

Studies of determinants of adherence identified being female [29, 33, 35, 38, 41], age over 50 years [29, 33], not living alone [29, 38], employment [27, 33], higher education [33], and living in a city [41], comorbidity including: ischemic heart disease [24, 29, 38], history of myocardial infarction [26, 29], arrhythmias [27], and diabetes mellitus [24, 29, 35]. These are consistent with previous systematic review of studies from elsewhere [44]. Data of association with anxiety level, panic attacks and subclinical depression [19] differ from those in the publication [44].

Evidence for an association between eligibility for the MAS, grade of HT, and experience of hypertensive crisis was inconclusive [24, 27, 29, 33, 38–41]. In two [29, 40], patients with a higher BP were more adherent and in another two [27, 33] better adherence was observed in patients with a lower grade of HT. In one [41], patients who had experienced a hypertensive crisis had higher adherence but in another [39] adherence was lower. There were also conflicting findings on associations with duration of HT. In three [29, 38, 40] patients with a long history of HT were more adherent but the reverse was observed two [27, 39]. One reason for such discrepancies could be heterogeneity of patients included. In addition, sample sizes varied greatly, with 1419 [38, 39], *n* = 161 [27], *n* = 190 [41] and *n* = 30 [40]. There was no evidence that adherence was associated with income and physical activity [29].

Almost all interventions studies found significant results, possibly reflecting publication bias. They included optimization of the drug regimen [11, 15, 16, 18, 19, 32, 33, 35, 36], an educational program [12, 13, 19–23], provision of an automatic home BP monitor [31, 37], and an initiative to inform the patient of his or her risk [14].

Provision of an automatic BP monitor [31] and optimization of the drug regimen using fixed combinations [11, 15, 16, 18, 19, 32, 33, 35, 36] found to be effective elsewhere [45, 46], were associated with a significant increase in adherence to AHT. However, in several of the studies adherence also improved in control groups, most likely because both groups received a series of enhancements to treatment including self-monitoring diaries, and written recommendations on lifestyle changes, as well as intensive monitoring of both groups for the entire period of follow-up. Thus, two of the randomized controlled studies found no differences between intervention and control groups, in adherence or BP reduction [31, 32]. The authors of two studies [11, 15] concluded that the results support fixed-dose combinations, but this seemed difficult to justify from their findings.

### Strengths and limitations

The randomized trials included, none of which were blinded, had a high risk of systemic error (bias), while using questionnaires that subjectively measure adherence and reporting disparate numbers of patients in the intervention and control groups [37], including only non-adherent patients [12, 13], and in two randomized non-blinded controlled trials no baseline adherence rates were reported (*incomplete data presentation)* [14, 37]. As a consequence, the risk of a systemic error in our review is close to critical.

In the vast majority of studies, the antihypertensive effect of adherence was measured as mean BP or probability of achieving target BP levels. Only one study measured 24-h BP [30] or used home BP monitoring [37].

Most studies used subjective methods of assessment, namely questionnaires, including some developed by the authors themselves, with no information on their testing or validation. The only objective method used for assessing adherence was indirect, using pill counts.

This situation is regrettable, because other methods for assessing adherence exist, differing in the degree of objectivity and information provided. The subjective methods include, first, various validated questionnaires, among which the MMAS-4 is most often used [47]. Subjective methods of assessing adherence, based on a patient’s self-assessment, should be used with caution. Adherence rates are overestimated by up to 20% compared with an objective assessment [47]. Objective indirect methods of assessing adherence include pill counts, as well as various electronic dispensers [48]. Electronic prescription and claims data on medication dispensed has also been used to evaluate an adherence, assessed by the proportion of days covered, although obviously it assumes that medicines dispensed are actually taken [49, 50]. Abroad, a way of controlling adherence is available, such as the analysis of electronic databases of pharmacy chains [51]. The “gold standard” for assessing adherence is measurement of drugs in biological fluids, for example, in blood or urine, with the latter preferred as it is less invasive [52].

Our findings suggest that interest in this issue is increasing among Russian researchers. There were 85% more publications in the period 2013 to 2017 than in the previous five-year period. However, the methodological quality of the papers has not improved. There is no obvious improvement in study design, with no more randomized trials and although the MMAS-4 questionnaire has been used more often, the recent increase in studies cannot be considered a major achievement.

Notwithstanding the many methodological weaknesses, these findings suggest a picture of unsatisfactory adherence to drug treatment in the Russian hypertensive population. However, the quality of studies of adherence to AHT is a serious problem not only in Russia, but also internationally. The 2014 Cochrane review of interventions to improve adherence [53] found only 13 studies on AHT that were suitable for inclusion. It noted the unsatisfactory (poor) overall level (quality) of such studies and emphasized the need to use objective indicators of adherence. For this purpose, Ascertaining Barriers for Compliance project was developed [54], as well as Emerge’s recommendations, that was published in order to solve the lack of standard methods to assess adherence [55].

There were the limitations of the scoping review process, e.g. last search dated was 2017, and reports published only in the Russian language.

Despite the limitations of most of the studies included this review makes a contribution in the following respects.

First, it is the first attempt to scope a comprehensive picture of the Russian literature on this topic. An important contribution of this paper is that it captures the full spectrum of research on adherence in Russia. It has identified some large studies, with prolonged follow up, using internationally accepted measures. Collectively, the small number of better quality studies does offer insights that can help inform the design of relevant policies, although further evidence is essential. Adherence to AHT in Russia is clearly a problem. Given the considerable economic burden that this creates, borne by both patients themselves and the health system as a whole, this is an issue that should be considered a high priority.

Second, the evaluative studies do point to some potentially promising measures. However, all should be subject to further evaluation and there is a clear need for much more research on interventions that have been found to be promising elsewhere [56–58].

## Conclusions

The main determinants of adherence to AHT are sociodemographic factors, such as female gender, age over 50 years, not living alone, employment, higher education, and living in a city; comorbidity, including: ischemic heart disease, history of myocardial infarction, arrhythmias, diabetes mellitus, and psychiatric disorders; adherence was also associated with onset of HT at a young age and use of fixed dose combination therapy. The findings of studies of the association between adherence and grade of HT, experience of hypertensive crisis, and duration of HT were not consistent. Patient education, telephone reminders, home BP monitoring and fixed-dose combinations of drugs are most important for improving adherence. The interpretations of these findings are limited by unreliable measures of adherence. It is necessary to introduce objective methods for assessing of adherence. A central repository of studies published in regional medical journals should be created.

## Additional files

Additional file 1: Preferred Reporting Items for Systematic reviews and Meta-Analyses extension for Scoping Reviews (PRISMA-ScR) Checklist. (DOCX 26 kb)

Additional file 2: Full search strategies on adherence to antihypertensive therapy in adult population in Russia from 2000 to 2017. (DOCX 17 kb)

Additional file 3: Levels of adherence in prevalence studies or baseline of interventions in adult population with hypertension in Russia from 2000 to 2017. (DOCX 22 kb)

Additional file 4: Sociodemographic and clinical factors associated with adherence to antihypertensive therapy in adult population with hypertension in Russia from 2000 to 2017. (DOCX 30 kb)

Additional file 5: Effectiveness of interventions aimed at increasing adherence in adult population with hypertension in Russia from 2000 to 2017. (DOCX 23 kb)

## Data Availability

All data generated or analysed during this study are included in this published article and its additional files.

## Abbreviations

AHT: Antihypertensive therapy
BP: Blood pressure
HT: Hypertension
MAS: Medicine Assistance Scheme
MMAS-4: 4-item Morisky Medication Adherence Scale
PRISMA-ScR: Preferred Reporting Items for Systematic reviews and Meta-Analyses extension for Scoping Reviews
SCORE: Systematic Coronary Risk Evaluation scale
