# Supplementary material for: Adherence to antihypertensive medication in Russia: a scoping review of studies on levels, determinants and intervention strategies published between 2000 and 2017
Source: Arch Public Health. 2019 Sep 25;77:43. doi: 10.1186/s13690-019-0366-9 (PMC6760051; doi:10.1186/s13690-019-0366-9)
Supplement: Supplementary file 2 — Full search strategies on adherence to antihypertensive therapy in adult population in Russia from 2000 to 2017. (DOCX 17 kb) [file 13690_2019_366_MOESM2_ESM.docx]

**Additional file 2.** Full search strategies on adherence to antihypertensive therapy in adult population in Russia from 2000 to 2017

Search strategy used for eLIBRARY.ru

The initial search

«Что искать» (формула поиска): комплае* OR комплай* OR приверженност* OR complianc* OR noncomplianc*

«Где искать»: в названии публикации, в аннотации, в ключевых словах;

«Тип публикации»: статьи в журналах, книги, материалы конференции, депонированные рукописи, диссертации, отчеты, патенты.

English translation:

"What to looking for"(search formula): compliance* OR adherence*;

"Where to looking for": in title, abstract, or keywords;

"Type of publication": articles in journals, books, conference materials, deposited manuscripts, dissertations, reports, patents.

The second search conducted "among the initial results was"

«Что искать»: антигипертенз* OR гипертенз* OR гипертон*OR гипотензивн*;

«Где искать»: в названии публикации, в аннотации, в ключевых словах.

«Тип публикации»: статьи в журналах, книги, материалы конференции, депонированные рукописи, диссертации, отчеты, патенты;

«Параметры»: искать в результатах предыдущего запроса.

English translation:

"What to looking for"(search formula): antihypertensives* OR hypertensives* OR hypertones* OR hypotensive*;

"Where to looking for": in title, abstract, or keywords;

"Type of publication": articles in journals, books, conference materials, deposited manuscripts, dissertations, reports, patents;

"Parameters": search in the results of the previous query.

Search strategy used for Central Scientific Medical Library "Russian Medicine"

((((FT *комплае*) OR (FT *комплай*) OR (FT *приверженност*) OR (FT *кооперативност*) OR (FT *COMPLIANC*) OR (FT *NONCOMPLIANC*) OR (FT *COMPLIANT*) OR (FT *NONCOMPLIANT*) OR (FT * ADHERENCE *) OR (FT * NONADHERENCE *) OR (FT * ADHERENT*) OR (FT * NONADHERENT *) OR (FT * ADHERENCE *))) AND (((FT антигипертенз*) OR (FT гипертенз*) OR (FT гипертон*))))

English translation: compliance; noncompliance; compliant; noncompliant; adherence; nonadherence; adherent; nonadherent; antihypertensive; hypertensive; hypertension.

Search strategy used for Embase

#1 russia*

#2 antihypertens*

#3 "patient compliance" OR adherence OR russia:ca

#4 #1 AND #3

#5 #2 AND #4

Search strategy used for PubMed

PubMed was searched for references containing the words “drugs“ AND “increase“ OR “decrease“ AND “compliance“ OR “adherence“ AND “hypertension“ AND “Russia“.

Search details used for PubMed

("pharmaceutical preparations"[MeSH Terms] OR ("pharmaceutical"[All Fields] AND "preparations"[All Fields]) OR "pharmaceutical preparations"[All Fields] OR "drugs"[All Fields]) AND increase[All Fields] OR decrease[All Fields] AND ("patient compliance"[MeSH Terms] OR ("patient"[All Fields] AND "compliance"[All Fields]) OR "patient compliance"[All Fields] OR "compliance"[All Fields] OR "compliance"[MeSH Terms]) OR adherence[All Fields] AND ("hypertension"[MeSH Terms] OR "hypertension"[All Fields]) AND ("russia"[MeSH Terms] OR "russia"[All Fields])
