# Supplementary material for: Adherence to antihypertensive medication in Russia: a scoping review of studies on levels, determinants and intervention strategies published between 2000 and 2017
Source: Arch Public Health. 2019 Sep 25;77:43. doi: 10.1186/s13690-019-0366-9 (PMC6760051; doi:10.1186/s13690-019-0366-9)
Supplement: Supplementary file 3 — Levels of adherence in prevalence studies or baseline of interventions in adult population with hypertension in Russia from 2000 to 2017. (DOCX 22 kb) [file 13690_2019_366_MOESM3_ESM.docx]

**Additional file 3.** Levels of adherence in prevalence studies or baseline of interventions in adult population with hypertension in Russia from 2000 to 2017

| **Reference** | **Adherence measure** | **MMAS-4 score, points** | **MMAS-4 (% achieving score of 4)** | **Other measure** |
| --- | --- | --- | --- | --- |
| Ageev et al. [11] | MMAS-4 | 1.62 ± 0.27 (intervention group)  1.75 ± 0.31 (control group) | 15.4% (intervention group)  16.7% (control group) | - |
| Fofanova et al. [15] | MMAS-4 | 2.11±0.22 (intervention group)  2.64±0.26 (control group) | 11.1% (intervention group)  28,0% (control group) | - |
| Karpov et al. [16] | MMAS-4 | 2.78±1.39 | 45,05% | - |
| Smirnova et al. [31] | MMAS-4, pill counts | 1.7±1.2 (intervention group)  1.3±1.4 (control group) | - | According to pill counts compliance 7% (intervention group), 11% (control group) |
| Glezer et al. [17] | MMAS-4 | 2.76±1.25 | no data | - |
| Glezer et al. [18] | MMAS-4 | 2.95±1.22 | no data | - |
| Glezer et al. [19] | MMAS-4 | 2.2 | no data | - |
| Chazova et al. [23] | MMAS-4 | 1.8±0.9 (intervention group)  1.9±0.9 (control group) | no data | - |
| Kaskaeva et al. [21] | MMAS-4 | no data | 1 group (train drivers) 9.8±2.8%  2 group (other railway workers) 14.0±4.9%  3 group (non-railway workers) 38.6±5.2% | - |
| Fofanova et al. [24] | MMAS-4 | no data | 1871 out of 4816 ppl (38.8%). Calculated from the reports data | - |
| Donirova et al. [25] | MMAS-4 | no data | 14 out of 74 ppl (19%).  Calculated from the reports data | - |
| Loukianov et al. [26] | MMAS-4 | no data | 30.6% (patients without history of MI),  37.2% (patients with history of MI) | - |
| Fofanova et al. [27] | MMAS-4 | no data | 30 out of 161 ppl (19%). Calculated from the reports data | - |
| Sviryaev et al. [33] | MMAS-4, pill counts | - | 38% | no data |
| Olejnikov et al. [30] | MMAS-4 | no data | 20% (with 3 or 4 points) | - |
| Kotovskaya et al. [35] | MMAS-4 | 3.12±0.86 | 44.2% | - |
| Kagramanyan [20] | Bespoke questionnaire | - | - | 27% of ppl are adherent |
| Ushakova et al. [22] | Bespoke questionnaire | - | - | 38.5% of ppl are adherent |
| Soboleva et al. [28] | Bespoke questionnaire | - | - | 56% of ppl are adherent |
| Oganov et al. [29] | 1 question about the regularity of taking drugs | - | - | 60.5% of ppl are adherent (1510 out of 2496 ppl). Calculated from the reports data |
| Vologdina et al. [32] | Pill counts | - | - | According to pill counts compliance 43% (1 group, comb. therapy),  48% (2 group, 2 drugs separately) |
| Kontsevaya et al. [38] | Bespoke questionnaire | - | - | 74.9% of ppl are adherent |
| Panov et al. [36] | Bespoke questionnaire, pill counts | - | - | There are no basic data, it is only results of adherence assessment in 6 and 12 months after intervention |
| Kopnina et al. [40] | Bespoke questionnaire | - | - | 23.3% of ppl are adherent |
| Sergeeva [41] | Bespoke questionnaire | - | - | 38% high level  42% middle  20% low level |
| Oschepkova et al. [37] | Bespoke questionnaire | - | - | Regularly took of prescribed medications: 84% of patients using home BP monitoring and 27% - in comparison group |
| Kobalava et al. [12, 13] | MMAS-4 | Included only insufficiently adherent patients having less than 4 points on MMAS-4 at baseline | | - |

HT, arterial hypertension; MMAS-4, 4-item Morisky Medication Adherence Scale; ppl, people; * – MMAS-4 with interpretation of results different from the original version.
