# Supplementary material for: Adherence to antihypertensive medication in Russia: a scoping review of studies on levels, determinants and intervention strategies published between 2000 and 2017
Source: Arch Public Health. 2019 Sep 25;77:43. doi: 10.1186/s13690-019-0366-9 (PMC6760051; doi:10.1186/s13690-019-0366-9)
Supplement: Supplementary file 4 — Sociodemographic and clinical factors associated with adherence to antihypertensive therapy in adult population with hypertension in Russia from 2000 to 2017. (DOCX 30 kb) [file 13690_2019_366_MOESM4_ESM.docx]

**Additional file 4.** Sociodemographic and clinical factors associated with adherence to antihypertensive therapy in adult population with hypertension in Russia from 2000 to 2017

| **Reference** | **Adherence measure** | **Factor associated with adherence (reference category none unless otherwise stated)** | **Univariate or multivariate** | **Main findings** | **P** |
| --- | --- | --- | --- | --- | --- |
| Fofanova et al. [24] | MMAS-4 | IHD | Univariate | Adherent: 49.4% had IHD / non adherent: 43.3% had IHD | 0.01 |
|  |  | DM |  | Adherent: 23.7% had DM / non adherent: 13.8% had DM | 0.001 |
|  |  | Eligibility for the MAS |  | Adherent: 43.9% be able to use MAS / non adherent: 56.1% be able to use MAS | 0.001 |
|  |  | Home BP device |  | Adherent: 78.3% home BP device is available / non adherent: 67.9% home BP device isn’t available | 0.001 |
| Morozov et al. [34] | MMAS-4*, pill counts | More frequent visits to the doctor | Univariate | Adherent: pts who visited doctor every month had 0.43±0.12 points, compliance rates 87% / non adherent: pts who visited doctor once a year had 2.32±0.72 points, compliance rates 61% | < 0.01 |
| Kotovskaya et al. [35] | MMAS modified | Female | Univariate | Female had 3.21±0.77 points / male had 3.06±0.71 points | <0.05 |
|  |  | DM |  | Pts with DM had 3.32±0.71 points / pts without DM had 3.14±0.70 points | <0.05 |
|  |  | Intake only of one antihypertensive drug |  | Pts taking 1 drug had 3.41±0.74 points / pts taking 3 drugs had 3.09±0.76 points | <0.05 |
|  |  | Intake only of two antihypertensive drugs |  | Pts taking 2 drugs had 3.27±0.79 points / pts taking 3 drugs had 3.09±0.76 points | <0.05 |
| Sviryaev et al. [33] | MMAS-4, pill counts | Female (*vs* male) | Univariate | χ^2^=78.43  There are no numerical values of adherence level | <0.001 |
|  |  | Lower HT grade |  | Lower HT grade is associated with high adherence, χ^2^=63.38. There are no numerical values of adherence level | <0.001 |
|  |  | Age 50 years and older |  | Age ≥50 years is associated with high adherence, χ^2^=68.29.  There are no numerical values of adherence level | <0.001 |
|  |  | Not living alone |  | Not living alone is associated with high adherence, χ^2^=70.39.  There are no numerical values of adherence level | <0.001 |
|  |  | Employed (vs unemployed) |  | Employed have higher adherence, χ^2^=56.98. There are no numerical values of adherence level | <0.001 |
|  |  | Higher education (*vs* less than higher education) |  | Higher education is associated with high adherence, χ^2^=65.44.  There are no numerical values of adherence level | <0.001 |
| Donirova et al. [25] | MMAS-4 | Female | Univariate | Adherent: 78.5% were female / non adherent: 66.7% were female | ns |
|  |  | Age 51-60 years |  | Adherent: 42.8% had age 51-60 years / non adherent: 33.3% had age 51-60 years | ns |
|  |  | Employment |  | Adherent: 57.1% were employed / non adherent: 65.0% were employed | ns |
| Kontsevaya et al. [38] | 1 question about the regularity of taking drugs | Female (*vs* male) | Univariate | Non adherent: 29.9% male *vs* 23.0% female | <0.001 |
|  |  | HT duration |  | Non adherent: less than 5 years – 31.3% *vs* more than 5 years 19.0% | <0.001 |
|  |  | IHD |  | Non adherent: 19.3% had IHD *vs* 31.7% had not IHD | <0.01 |
|  |  | Eligibility for the MAS |  | Non adherent: 19.1% be able to use MAS *vs* 30.2% not be able to use MAS | <0.001 |
|  |  | Use of fixed combinations |  | Non adherent: 18.9% didn`t use of fixed combinations *vs* 13.5% use of fixed combinations | <0.05 |
| Kontsevaya et al. [39] | 1 question about the regularity of taking drugs | Female (*vs* male) | Univariate | Non adherent: 75.6% male *vs* 74.6% female | ns |
|  |  | HT duration |  | Non adherent: more than 5 years 76.5% *vs* less than 5 years 68.9% | <0.001 |
|  |  | Eligibility for the MAS |  | Non adherent: 82.9% be able to use MAS *vs* 67.3% not be able to use MAS | <0.001 |
|  |  | Hypertensive crisis |  | Non adherent: 81.5% had history of crisis *vs* 70.3% hadn`t history of crisis | <0.01 |
|  |  | Number of antihypertensive drugs |  | Non adherent: 79.6% pts taking 3 or more drugs *vs* 61.8% pts taking 1-2 drugs | <0.001 |
| Kopnina et al. [40] | Bespoke questionnaire | HT duration | Univariate | Adherent: HT duration was 17.3±2.88 yrs / non adherent: HT duration was 8.7±2.39 yrs | < 0.05 |
|  |  | Higher level of SBP |  | Adherent: pts had the SBP level 173±5.6 mm Hg / non adherent: pts had the SBP level 150±2.86 mm Hg | < 0.05 |
| Loukianov et al. [26] | MMAS-4 | History of MI | Univariate | Adherent: 37.0% had MI in anamnesis / non adherent: 30.6% had MI in anamnesis | 0.03 |
| Sergeeva et al. [41] | Bespoke questionnaire  (adherence is rated in points:  high adherence corresponds to 12 points or more, moderate - 8-11 points, low - 7 points and less) | Female | Univariate | Female are more adherent, than men: 11.8±0.5 *vs* 8.9±0.6 points | <0.05 |
|  |  | Living in the city |  | Those living in a city are more adherent, than living in a village 10.2±0.7 *vs* 8.7±0.4 points | <0.05 |
|  |  | Hypertensive crisis |  | Patients the having hypertensive crises are more adherent, than not having: 12.3±0.8 *vs* 10.16±0.4 points | <0.05 |
| Fofanova et al. [27] | MMAS-4 | Employment | Univariate | Adherent: 90% were employed / non adherent: 61% were not employed | 0.003 |
|  |  | Concomitant diseases |  | Adherent: 15.3% had concomit diseases / non adherent: 6.9% had concomit diseases | <0.05 |
|  |  | Higher level of SBP |  | Adherent pts had the SBP level 147.3±3.6 mm Hg / non adherent: had the SBP level 155.2±2.6 mm Hg | <0.05 |
|  |  | HT duration |  | Adherent: HT duration was 7.4±1.7 yrs / non adherent: HT duration was 11.2±1.4 yrs | <0.05 |
|  |  | Age of onset of HT |  | Adherent: age of onset of HT 38.1±1.5 yrs / non adherent: age of onset of HT 44.3±2.4 yrs | <0.05 |
|  |  | Dysthymia |  | Adherent: 40% had dysthymia / non adherent: 6.9% had dysthymia | <0.00001 |
|  |  | Panic attacks |  | Adherent: 56.7% had panic attacks / non adherent: 7.6% had panic attacks | <0.00001 |
|  |  | High anxiety level and subclinical depression |  | Adherent: 23.3% had high anxiety level and subclinical depression / non adherent: 3.8% had high anxiety level and subclinical depression | 0.0002 |
| Oganov et al. [29] | 1 question about the regularity of taking drugs | Age 60 years and older | Multivariate and univariate | Adherent: 57.0% was aged 60 yrs and older / non adherent: 48.35% was aged 60 yrs and older | <0.001 |
|  |  | Gender (m/f) |  | Male/female OR 0.79 (95% CI 0.67-0.94)  Adherent:m/f was 29.01/70.99% / non adherent: m/f was 34.08/65.92% | 0.007  <0.01 |
|  |  | Marital status |  | Adherent: 8.91% of pts divorced / non adherent: 11.59% of pts not divorced | <0.001 |
|  |  | HT duration |  | Adherent: HT duration was 14.27±9.64 yrs / non adherent: HT duration was 11.86±9.44 yrs | <0.001 |
|  |  | Grade 3 of HT |  | Adherent: 33.20% had grade 3 of HT / non adherent: 21.57% had grade 3 of HT | <0.001 |
|  |  | IHD |  | Presence of IHD: OR 1.63 (95% CI 1.39-1.92)  Adherent: 59.15% had IHD / non adherent: 46.99% had IHD | 0.001  <0.001 |
|  |  | History of MI |  | Adherent: 13.91% had history of MI / non adherent: 9.13% had history of MI | <0.05 |
|  |  | DM |  | Adherent: 17.36% had DM / non adherent: 10.89% had DM | <0.001 |
|  |  | Home BP device |  | Possession of a home BP device: OR 2.81 (95% CI 2.15-3.67)  Adherent: 94.1% had home BP device / non adherent: 85.03% % had home BP device | 0.01  <0.001 |
|  |  | Low income |  | Adherent: 41.57% had low income / non adherent: 44.34% % had low income | ns |
|  |  | Low physical activity |  | Adherent: 17.6% had low physical activity / non adherent: 19.3 % had low physical activity | ns |

BP, blood pressure; DM, diabetes mellitus; HT, arterial hypertension; IHD, ischemic heart disease; MAS, medicine assistance scheme; MI, myocardial infarction; MMAS-4, 4-item Morisky Medication Adherence Scale; ns, not significant; SBP, systolic blood pressure; * – MMAS-4 with interpretation of results different from the original version; pts, patients.
