# Supplementary material for: Adherence to antihypertensive medication in Russia: a scoping review of studies on levels, determinants and intervention strategies published between 2000 and 2017
Source: Arch Public Health. 2019 Sep 25;77:43. doi: 10.1186/s13690-019-0366-9 (PMC6760051; doi:10.1186/s13690-019-0366-9)
Supplement: Supplementary file 5 — Effectiveness of interventions aimed at increasing adherence in adult population with hypertension in Russia from 2000 to 2017. (DOCX 23 kb) [file 13690_2019_366_MOESM5_ESM.docx]

**Additional file 5.** Effectiveness of interventions aimed at increasing adherence in adult population with hypertension in Russia from 2000 to 2017

| **Reference** | **Design** | **Sample** | **Age** | **HT grade** | **Duration** | **Nature of intervention** | **Adherence measure** | **Main findings,**  **points according to MMAS-4,**  **% of adherent** |
| --- | --- | --- | --- | --- | --- | --- | --- | --- |
| Vologdina et al. [32] | Randomized intervention study | 70 | 80.7± 2.7 (female)/  80.3±2.5 (male) | 1-2 | 3 mth | FC amlodipine/  lisinopril | Pill counts | Increased of compliance rate from 43% to 84%, р<0.0001 |
| Smirnova et al. [31] | Randomized intervention study | 60 | 45-75 y | 1-2 | 3 mth | Provision of home BP monitoring | MMAS-4,  pill counts | Increased score in intervention group: from 1.7±1.2 to 3.0±1.1, р=0,000;  compliance rates according to pill counts: from 5% to 96,4%, p<0.001 |
| Oschepkova et al. [37] | Randomized intervention study | 30 | 30-71 y | 1-2 | 12 mth | BP self-monitoring, patient education | Bespoke questionnaire | Intervention group - 84% of adherent ppl *vs* 27% in control group, p<0.05 |
| Ageev et al. [11] | Randomized intervention study | 60 | 62.5±2.2 | 1-2 | 6 mth | FC enalapril/  hydrochlorothiazide | MMAS-4 | Increased score in intervention group: from 1.62±0.27 to 3.53±0.21, р=0.00001. There is no difference between the intervention and control groups |
| Kobalava et al. [12, 13] | Randomized intervention study | 906 | 56.2±10.6 (female)/  54.9±10.9 (male) | Uncontrolled HT | 12 mth | Free first package of perindopril + patient education+ phone reminders | MMAS-4 | Proportion of adherent ppl in intervention and control groups: 52.2% *vs* 71.7%, p<0.0001 |
| Sarycheva et al. [14] | Randomized intervention study | 150 | 40-65 y | no data | 12 mth | Electronic version of the SCORE | MMAS-4 | 6 months: points in intervention and control group:2.75 *vs* 1.88, p<0.001;  12 months: points in intervention and control group: 2.14 *vs* 1.27, р<0.001 |
| Fofanova et al. [15] | Randomized intervention study | 60 | 61.2±1.8 (female)/  61.8 ±2.1 (male) | 1-2 | 6 mth | Fixed in one blister combination of enalapril/ indapamide | MMAS-4 | Increased points in intervention group: from 2.11±0.22 to 3.37±0.19, р=0.00006; Increased % of ppl with 4 points in intervention group: from11.1 to 59.3, р=0.0003 |
| Kotovskaya et al. [35] | Prospective observational intervention study | 2435 | 59.3±11.2 | Uncontrolled HT | 3 mth | FC perindopril/  amlodipine | MMAS modified | Increased points in intervention group from 3.12±0.86 to 3.6±0.74, p<0.05.Increased % of ppl with 4 points: from 44.2% to 82.8%, p – no data. |
| Karpov et al. [16] | Prospective observational intervention study | 2120 | 22-88 y | 2-3 | 3 mth | FC perindopril/ indapamide + more frequent visits to the doctor | MMAS-4 | Increased points from 2.78±1.39 to 3.61±0.77, р=0.00001 |
| Glezer et al. [17] | Prospective observational intervention study | 957 | 56.5±11.5 | 1-2 | 3 mth | New drug form: orally disintegrating tablet of perindopril arginine | MMAS-4 | Increased points from 2.76±1.25 to 3.57±0.89, р<0.00001 |
| Glezer et al. [18] | Prospective observational intervention study | 1351 | 59.4±11.1 | Essential | 3 mth | FC perindopril/  amlodipine | MMAS-4 | Increased points from 2.95±1.22 to 3.59±0.94, p<0.05 |
| Glezer et al. [19] | Prospective observational intervention study | 1969 | 60.1±0.3 | no data | 3 mth | FC perindopril/ indapamide + patient education | MMAS-4 | Increased points from 2.80 to 3.79, p<0.0001 |
| Sviryaev et al. [33] | Prospective observational intervention study | 115 | 51.3±9.6 | 1-2 | 6 mth | FC enalapril/ indapamide in one blister + free first package | MMAS-4,  pill counts | Increased proportion of ppl with 4 points from 38% to 90%, р<0.001;  Increased of compliance rate to 97% |
| Kagramanyan [20] | Prospective observational intervention study | 50 | 64.06±0.49 (female)/  61.88± 1.28 (male) | 1-3 | 6 mth | Patient education | Bespoke questionnaire | Increased % of adherent ppl from 27% to 67%, p<0.05 |
| Kaskaeva et al. [21] | Prospective observational intervention study | 250 | 20- 64 y (male) | 1-3 | 6 mth | Patient education | MMAS-4 | Increased proportion of adherent ppl from 38.6±5.2% to 57.7±5.3%, р=0.04 |
| Panov et al. [36] | Prospective observational intervention study | 60 | 57.65±1.59 | 1-2 | 12 mth | FC amlodipine/lisinopril + free package + more frequent visits to the doctor | Bespoke questionnaire, pill counts | Proportion of adherent ppl in intervention and control group: 93% *vs* 80%, p value - no data;  pill counts - no data |
| Ushakova et al. [22] | Prospective observational intervention study | 52 | 50.08±7.25 | 2 | 6 mth | Patient education | Bespoke questionnaire | Increased proportion of daily taking drugs ppl from 38.5% to82.7%, р<0.05. Increased readiness of patients to pay money for antihypertensive drugs from 230±199.6 to 327±272 rubles, р<0.05. |
| Chazova et al. [23] | Prospective observational intervention study | 193 | 60.3±8.0 | no data | 6 weeks | Patient education | MMAS-4 | Increased points in intervention group: from 1.8±0.9 to 3.9±0.1, р=0.0002 |

BP, blood pressure; FC, fixed-dose combination; HT, arterial hypertension; MMAS-4, 4-item Morisky Medication Adherence Scale; ppl, people; mth, months.
